# A twenty-year follow-up of canine leishmaniosis in three military kennels in southeastern France

**DOI:** 10.1186/1756-3305-6-323

**Published:** 2013-11-09

**Authors:** Bernard Davoust, Cédric Roqueplo, Daniel Parzy, Stéphanie Watier-Grillot, Jean-Lou Marié

**Affiliations:** 1 Animal Epidemiology Working Group of the Military Medical Service, Toulon, France; 2 Research Unit of Emerging Infectious and Tropical Diseases (URMITE) UMR CNRS, 7278 IRD 198 INSERM 1095, Aix-Marseille-Université, Marseille, France; 3 UMR- MD3, Faculté de pharmacie, Aix-Marseille-Université, Marseille, France; 4 Institut de recherche biomédicale des armées, Brétigny, France

**Keywords:** Canine leishmaniosis, Indirect fluorescent antibody test, *Leishmania infantum*, Seroprevalence, France, Deltamethrin, Dog

## Abstract

**Background:**

Canine leishmaniosis (CanL) is enzootic in southeastern France, and military working dogs (MWD) posted in this area are highly exposed. To assess the efficiency of prevention, we performed a serological and clinical follow-up of 80 MWD in the Var and Corsica regions during the 20-year period from 1993 to 2012. The systematic and specific prevention of CanL using a deltamethrin-impregnated collar (DMC) was implemented in 2002.

**Findings:**

Out of 80 dogs tested annually, the cumulative serological and clinical incidence was 42.5% (34/80) and 21.2% (17/80) respectively, during the first period, and these numbers decreased to 6.2% (5/80) and 2.5% (2/80) during the second period (*p* < 0.00001). Considering the incidence of serology since 2002, the CanL risk has been reduced by 85.2% and the level of protection of MWD reached 93.8%. Dogs without collars had a 10.4-fold greater chance of becoming infected than protected dogs. Although other ecological factors might have influenced the epidemiology of CanL, DMC usage was the main factor affecting dog exposure to CanL.

**Conclusions:**

The prevention strategy based on DMC proved highly efficient in our population of MWD, as it effectively controlled the disease. This result is also of interest to public health, as dogs are reservoirs for *Leishmania infantum*.

## Findings

Canine leishmaniosis (CanL), caused by the protozoan *Leishmania infantum* Nicolle (family Trypanosomidae; class Kinetoplastida), has a high incidence in all Mediterranean regions and in many Latin American countries [1]. Dogs are the main reservoir of the parasite that causes human visceral leishmaniosis, a major zoonosis. Infection by *Leishmania* is almost exclusively vectorial, via the bite of female sandflies of the subgenus *Phlebotomus* (*Larroussus*) [1]. In France, CanL is primarily located on the Mediterranean coast but has expanded its distribution area to the north and east. The most affected area is the Andorra Nice-Lyon triangle [2]. Infections are seasonal, occurring from spring to autumn. The control of CanL is important for two reasons [3,4]. In the field of animal health, CanL is clinically characterized by mucocutaneous and visceral involvement, which gradually progresses towards the death of the animal [5]. There is also a public health issue. Traditionally affecting children, human leishmaniosis has become an opportunistic infection of immunocompromised adults [6]. Whilst treatments are effective they are also long, stressful and rather toxic. Consequently, exposed dogs must be treated with effective prophylactic measures.

In 1993, CanL seroprevalence was assessed using an indirect fluorescent antibody test (IFAT) in three kennels of military working dogs (MWD) in southeastern France. The cumulative seroprevalence was 11.6% (9/77), including 22.7% (5/22) in Solenzara (Corsica) and 7.2% (4/55) in Toulon/Hyères [7]. During the following 20 years, preventive measures and monitoring of leishmaniosis were implemented. The average global population was 80 dogs and yearly changes in the average were minor. We included Belgian shepherd malinois (for the majority) and German shepherd, males, aged 2–9 years, that originated from countries non-endemic for leishmaniosis (Belgium, Luxembourg, Netherlands and Germany). Since 2002, all MWD wore a 4% (40 mg/g) deltamethrin-impregnated collar (DMC) [Scalibor®, MSD International Animal Health] upon their initial arrival into CanL-enzootic areas. Collars were put in place each year in April and withdrawn in October. The dogs were working and living in three different military camps based near three cities in southern France: Toulon (longitude: 5°55’E; latitude: 43°10’N), Hyères (longitude: 06°09’E; latitude: 43°08’N) and Solenzara (longitude: 09°24’E; latitude: 41°55’N). Due to the proximity of Toulon and Hyères, they were merged into one area (Var), and Solenzara was considered a different area (Corsica). In addition, MWD in Toulon and Hyères belong to the same military unit and are regularly moved between kennels. These dogs do not leave the Var region. However, dogs in Solenzara have periodically completed short stays (four months) in Africa, the Balkans, Central Asia or French Guiana. All MWD were examined by veterinarians at least once a year during the vaccination boost (rabies and DHLPP). None of the MWD received vaccinations against CanL. A clinical diagnosis of CanL was made in the presence of the classical clinical signs (asthenia, weight loss, squamosis, alopecia, onychogryphosis, lymphadenomegaly, and nose .ulcers) associated with positive serology. Clinically ill MWD received a specific treatment (meglumine antimoniate and allopurinol). Dogs that were severely ill after treatments were euthanized.

At the beginning of each year, all dogs were subjected to a serological test (IFAT), but excluding the years: 1994, 1997, 2003, 2009 and 2011. Blood samples from the radial vein were centrifuged within 24 hours after collection, and the serum was stored at -20°C. IFAT is the reference test, sensitive and specific [1,8,9]. The *L. infantum* antigen (Synbiotics Laboratory, Lyon, France) consisted of formalized promastigotes. The antigen was added to immunofluorescent slides and fixed in cold acetone for 20 min. The collected serum was pre-diluted 20-fold and examined with an ultraviolet microscope at 500X magnification. The presence of anti-*Leishmania* antibodies was detected by promastigote fluorescence. Negative sera were not fluorescent. Titers ≥ 1:100 were considered positive, according to our laboratory reference. The correlation between DMC treatment and CanL incidence was evaluated with the Fischer test, the Mid-p exact test and the linear correlation test (Pearson’s coefficient) with a 95% significance level (*p* < 0.05) using Epi Info® and Open Epi® software.

**Figure 1:**
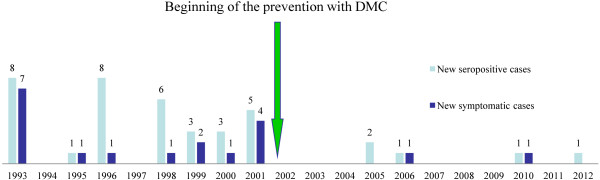
Incidence of leishmaniosis in a population of military dogs receiving a specific prevention (DMC) since 2002.

The longitudinal follow-up results from the canine population (average 80 MWD) are presented in Figure 1. There was no specific prevention of CanL for 9 years (1993–2001), and subsequently, DMC was used for 11 years (2002–2012). During this 20-year period, the number of MWD remained quite stable, but the individual dogs changed (transfer, discharge, death). However, the new replaced dogs were tested for CanL, and only seronegative dogs were introduced. On average, MWD stayed 7 years in the same kennel. In our study, all dogs with clinical CanL were seropositive. The incidence of CanL-seropositive dogs is summarized in Table 1. There was a significant difference between the period before 2002 (34 cases - 42.5%) and after 2002 (5 cases - 6.2%), although the second period is 2 years longer. Considering the incidence of positive serology since 2002, the CanL risk for these dogs was reduced by 85.2% and 93.8% of MWD were protected from disease. Based on the mean annual incidence, the relative risk was 8.4, which means that dogs without collars had an 8.4-fold greater chance of becoming seropositive than dogs protected by collars. Moreover, there was a significant difference in symptomatic dogs between these two periods: 17 clinical cases before 2002 and 2 afterwards (Table 2). Dogs without collars had a 10.4-fold greater chance of becoming clinically ill than protected dogs. Based on these data, the efficiency of prevention of clinical leishmaniosis was 88.2%. Due to a low number of dogs in Solenzara, the statistical test failed to find a significant difference. For both areas, half of the seropositive dogs were clinically ill. IFAT serological titers were higher before 2002, with 15 of 34 dogs exhibiting a titer ≥ 1:1600, but due to a low number of seropositive dogs after 2002, the difference is not significant (p = 0,058) (Table 3).

**Table 1:** Incidence of CanL seropositivity, before and after the use of DMC

**Locations**	**Number of dogs**	**Symptomatic dogs**		***p*-value**	**Power of the test**
		**1993-2001**	**2002-2012**		**(α = 0.05)**
Toulon/Hyères	46	25 (54.3%)	1 (2.1%)	< 0.00001	100%
Solenzara	34	9 (26.5%)	4 (11.8%)	> 0.1	30%
Total	80	34 (42.5%)	5 (6.2%)	< 0.00001	99.99%

**Table 2:** Incidence of morbidity due to CanL, before and after the use of DMC

**Locations**	**Number of dogs**	**Symptomatic dogs**		***p*-value**	**Power of the test**
		**1993-2001**	**2002-2012**		**(α = 0.05)**
Toulon/Hyères	46	10 (21.7%)	0 (0%)	< 0.001	92%
Solenzara	34	7 (20.5%)	2 (5.9%)	> 0.05	40%
Total	80	17 (21.2%)	2 (2.5%)	< 0.0001	97%

**Table 3:** Distribution of the serological titers

**Titers (IFAT)**	**Before 2002**	**After 2002**
1:100	6	
1:160		2
1:200	4	
1:320	2	
1:400	5	1
1:800	2	
1:1280		2
1:1600	15	
Total	34	5

We observed that wearing a DMC is significantly associated with a reduction of CanL incidence, but we could not conclude direct causality. In the absence of data from dogs in southeastern France, we contacted veterinary practices in Toulon, Hyères and Solenzara. In these areas, the results were identical; the number of CanL clinical cases had decreased, mainly since 2008. This reduction is attributed to the most frequent use of external antiparasitic products (personal communication). Moreover, in 2007–2008, we screened MWD for *Leishmania* using qPCR [10]. The prevalence was 66.6% (32/48) in the Var region and 22.6% (7/31) in Corsica.

Deltamethrin acts on the nervous membranes of phlebotomines, inducing a massive release of synaptic neurosecretions and neurohormones. Both anti-feeding and lethal effects are observed. Experimental trials have demonstrated that topical insecticides and DMC, can protect dogs from > 85% of sandfly bites for up to 6 months [11]. DMC reduces the contacts between dogs and phlebotomines, thus protecting dogs and as a result, humans, from visceral leishmaniosis.

Other studies have shown that DMC protects dogs in the field. In Tunisia, the efficiency was 100% in 42 treated dogs compared with a control group [12]. Of 60 dogs followed for two years in Italy, the efficiency was 51% [13]. In a study of 350 dogs in southern Italy, the efficiency was 46% during the first year and 86% in the second year [14]. This cumulative effect illustrates the epidemiological impact of DMC on vectorial transmission. In Brazil, 136 dogs were protected by DMC, resulting in an efficiency of 50% during a one-year period [15]. In Italy, the risk of CanL was reduced by 84% using DMC and also using a spot-on solution of permethrin [16]. In 2004, a spot-on treatment containing a combination of 10% imidacloprid and 50% permethrin was launched on the French market for the prevention of CanL (Advantix®, Bayer Animal Health). In experimental conditions, this spot-on has been successfully assessed to determine the number of sandflies (dead and alive) after treatment [17-19]. The results from field studies are also strongly positive and similar to those obtained with DMC [20]. In Italy, the efficiency fluctuated between 84 and 100% [16,21,22]. More recently, a collar with a combination of 10% imidacloprid and 4.5% flumethrin (Seresto®, Bayer Animal Health) was 100% efficient in the field [23].

In France, the annual number of human cases of visceral leishmaniosis (VL) reported to the national reference center was roughly unchanged until 2007, after which it decreased [24]. From 2001 to 2003, there were 66 human cases [8]. These cases mainly came from the well-known foci of Côte d'Azur, Provence and Cévennes; 40% of the patients were co-infected with HIV. These co-infections decreased after 2003 [24]. From 1999 to 2001 (3 years), 83 cases were reported, and there were only 149 cases for the period 2002–2010 (9 years). The difference between these 2 periods, which are included in our study, is statistically significant (*p* < 0.001) [24]. Thus, human leishmaniosis is in regression in southeastern France, due to lower infection pressure and fewer infected reservoir dogs; the dogs being better protected by adequate collars or spot-on treatments. The use of DMC or spot-on treatments is an interesting alternative to culling, which remains controversial [14,15].

## Conclusions

Serological and clinical surveillance of CanL was carried out in a population of 80 MWD during a 20-year period. The decrease in the incidence rate of symptomatic and asymptomatic cases is most likely multifactorial and related to the use of DMC and/or environmental changes. When preventive measures are not implemented, CanL rapidly spreads in kennels [25]. External antiparasitic products that are efficient against phlebotomines are essential for MWD in areas of France or abroad that are endemic for leishmaniosis. Vaccination should also be integrated into the prevention strategy, if necessary, and following the recommendations of experts [3,4]. Further epidemiological studies are required to investigate additional potential reservoirs (cats, horses, foxes, rodents), vector insecticide resistance, and the behavior of phlebotomines feeding on hosts.

## Abbreviations

CanL: Canine leishmaniosis; DMC: Deltamethrin-impregnated collars; IFAT: Indirect fluorescent antibody test; MWD: Military working dogs; VL: Visceral leishmaniosis.

## Competing interests

The authors declare that they have no competing interests.

## Authors’ contributions

BD organized the prevention measures and the epidemiosurveillance of CanL in French military kennels and wrote the manuscript. CR performed statistical analysis. DP participated in serological testing. SWG collected samples and clinical data. JLM participated in data analysis and revised the manuscript. All authors read and approved the final manuscript.
